# Drug-Induced QT Prolongation as a Result of an Escitalopram Overdose in a Patient with Previously Undiagnosed Congenital Long QT Syndrome

**DOI:** 10.1155/2014/917846

**Published:** 2014-07-01

**Authors:** Paul Singh, J. Martin Maldonado-Duran

**Affiliations:** ^1^Division of Maternal-Fetal Medicine, Department of Obstetrics and Gynecology, University of Missouri Kansas City School of Medicine, 2301 Holmes Street, Kansas City, MO 64108, USA; ^2^University of Missouri Kansas City School of Medicine, 2301 Holmes Street, Kansas City, MO 64108, USA

## Abstract

We present a case of drug-induced QT prolongation caused by an escitalopram overdose in a patient with previously undiagnosed congenital LQTS. A 15-year-old Caucasian female presented following a suicide attempt via an escitalopram overdose. The patient was found to have a prolonged QT interval with episodes of torsades de pointes. The patient was admitted to the telemetry unit and treated. Despite the resolution of the torsades de pointes, she continued to demonstrate a persistently prolonged QT interval. She was seen by the cardiology service and diagnosed with congenital long QT syndrome. This case illustrates the potential for an escitalopram overdose to cause an acute QT prolongation in a patient with congenital LQTS and suggests the importance of a screening electrocardiogram prior to the initiation of SSRIs, especially in patients at high risk for QT prolongation.

## 1. Introduction

Long QT syndrome (LQTS) is a disorder of myocardial conduction characterized by a prolonged QT interval seen on an electrocardiogram. LQTS can lead to a polymorphic ventricular tachycardia, known as torsades de pointes, which may subsequently lead to ventricular fibrillation and sudden cardiac death. Although often caused by mutations in genes that code for a variety of myocardial ion channels, LQTS may be caused by a variety of risk factors, including drug-induced side effects. Medications known to cause QT prolongation include quinolones, macrolides, class IA and class III antiarrhythmics, cholinergic antagonists, tricyclic antidepressants, and phenothiazines. Selective serotonin reuptake inhibitors (SSRIs) have also been shown to cause LQTS [1]. We describe a unique case of acute QT prolongation as a result of an escitalopram overdose in a patient that was eventually found to have a congenital LQTS.

## 2. Case Report

**Figure 1:**
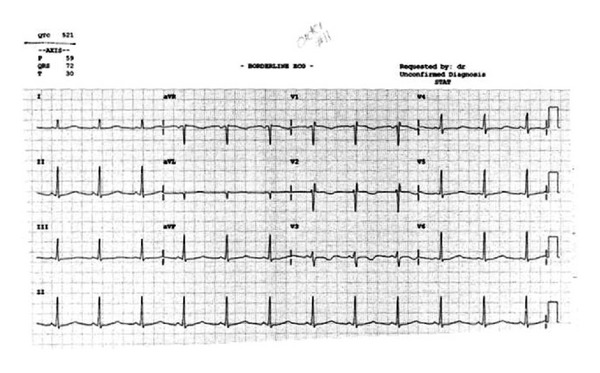
Initial electrocardiogram obtained in the emergency department. Note the prolonged QT interval of 521 ms.

**Figure 2:**
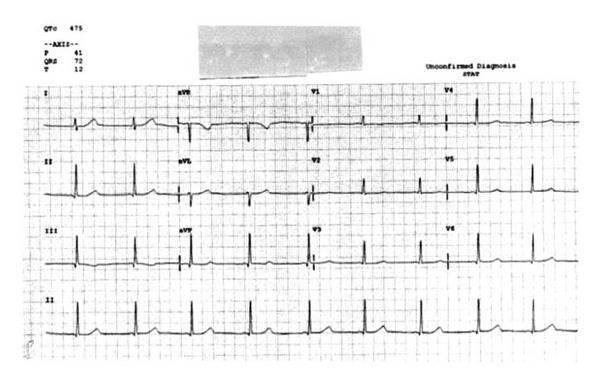
Electrocardiogram obtained on hospital day number 7. Note the persistently prolonged QT interval of 475 ms.

**Figure 3:**
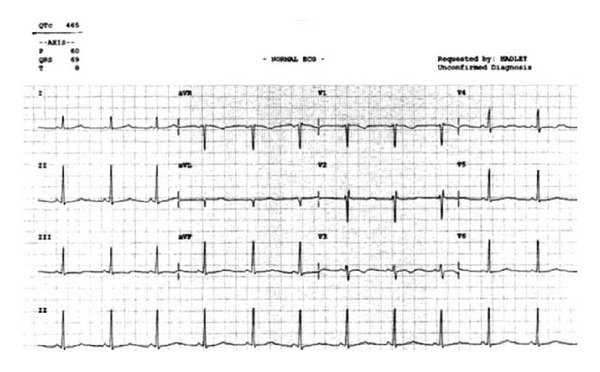
Electrocardiogram obtained 2 weeks after hospital discharge. Note the improved QT interval (465 ms) while on treatment for congenital QT prolongation.

A 15-year-old Caucasian female with a past medical history significant for depression presented to the emergency department following a suicide attempt after ingesting approximately 500 milligrams of escitalopram. She presented with lethargy and dizziness. Although her vital signs and physical examination were unremarkable, a prolonged QT interval of 521 milliseconds (Figure 1) along with multiple episodes of torsades de pointes was noted on the initial electrocardiogram. An initial supratherapeutic escitalopram level was found to be 350 ng/mL. The patient was diagnosed with drug-induced LQTS due to an escitalopram overdose and admitted to the telemetry unit for observation. Following treatment with magnesium sulfate and isoproterenol, the episodes of torsades de pointes resolved. Serial electrocardiograms, however, continued to demonstrate a prolonged QT interval. On the seventh hospital day the patient continued to demonstrate a prolonged QT interval of 475 milliseconds (Figure 2). By this time the escitalopram level had improved to 55 ng/mL. She was seen by the cardiology service and diagnosed with congenital LQTS. Unfortunately, the patient stated that she was adopted and, thus, could not provide a reliable family history of cardiac conduction abnormalities. She was started on propranolol and discharged home after being cleared by psychiatry. She was seen in the cardiology clinic two weeks after discharge and her QT interval had improved to 465 milliseconds (Figure 3). Molecular genetic testing performed on the patient revealed a KCNQ1 cardiac ion channel mutation.

## 3. Discussion

Previous estimates of the incidence of long QT syndrome (LQTS) have varied between 1/5000 and 1/10000. However, due to the increased number of cardiac ion channel mutations that have been recently identified, the incidence of LQTS may be higher [2]. Schwartz et al. reviewed nearly 45,000 neonates born in Italy and found that approximately 1/2500 were diagnosed with LQTS [2].

LQTS, short QT syndrome, sick sinus syndrome, catecholaminergic polymorphic ventricular tachycardia, early repolarization syndrome, and familial atrial fibrillation are all examples of congenital cardiac arrhythmias. An action potential is generated when the membrane is partially depolarized from the resting level to the threshold potential. The ensuing rapid depolarization is mediated by sodium entry into the cells due to an increase in the number of open sodium channels in the cell membrane. Repolarization results from potassium exit from the cells as the sodium channels are closed and potassium channels are opened. The QT interval, thus, represents the time interval between electrical depolarization and repolarization of the ventricles. In LQTS, it is hypothesized that derangements in cardiac ion flow lead to an increase in action potential duration. Specifically, prolongation of repolarization occurs as a result of a net reduction in the outward current mediated chiefly by decreased potassium efflux from the cardiac myocyte.

The QT interval is inversely influenced by the heart rate such that the faster the heart rate, the shorter the QT interval. A number of correction formulas have been developed in order to determine a corrected QT interval. The most utilized is Bazett's formula which is the measured QT interval divided by the square root of the RR interval [3]. Other alternative formulations for a corrected QT interval have also been proposed. Modern computer based ECG machines can easily determine and report a corrected QT interval. A borderline corrected QT interval in males ranges from 431 to 450 milliseconds and from 451 to 470 milliseconds in females, while a corrected QT interval over 450 ms in males and 470 ms in females is considered abnormal.

The molecular basis of congenital LQTS is heterogeneous and thus far thirteen cardiac ion channel genes have been described, each representing a separate subtype of congenital LQTS [4]. The most common mutations are KCNQ1, KCNH2, and SCN5A. Indeed, 90% of all known LQTS causal mutations are found in these three genes [4]. Mutations in the KCNQ1 gene result in the most common form of LQTS, referred to as LQTS type 1 (LQTS1). The KCNQ1 gene codes for a protein that mediates slowly activating current that accelerates the repolarization of action potentials in cardiac myocytes. The triggers for cardiac events in congenital LQTS are thought to be related to the underlying genotype. Schwartz et al. examined 670 patients with congenital LQTS and noted that the occurrence of a cardiac event differed according to genotype [5]. LQTS1 patients experienced the majority of cardiac events during exercise, while only 3 percent occurred at the rest. LQTS 2 and LQTS 3 patients, on the other hand, were less likely to have events during exercise and more likely to have events during rest [5].

In addition to congenital LQTS, LQTS may be induced by a variety of factors and substances. Risk factors for LQTS include electrolyte derangements, such as hypokalemia and hypomagnesemia, hypothyroidism, female gender, emotional stress, strenuous exercise activity, and bradycardia. A number of medications have been shown to cause a prolongation in the QT interval, including antihistamines, antiarrhythmics, decongestants, diuretics, antibiotics, and antidepressants. The susceptibility to develop an acquired LQTS may be influenced by the underlying genetic background [6]. Indeed, Itoh et al. demonstrated a similar mutation rate in the KCNH2, KCNQ1, and SCN5A cardiac conduction genes in acquired LQTS compared to congenital LQTS [6].

Depression is currently one of the fastest rising diagnoses made by office physicians. Visits for depression have nearly doubled since 1994. In addition, approximately 80% of physician visits for depression in 2004 resulted in a prescription for an antidepressant. Selective serotonin reuptake inhibitors (SSRIs) have been used for more than a decade for the treatment of depression and anxiety disorders. One advantage of this group of antidepressants is that they appear to be relatively safe when compared with older classes of antidepressants. Nevertheless, several cases related to resultant QT prolongation from overdoses of selective SSRIs such as fluoxetine, sertraline, and citalopram have been reported [1, 7]. In fact, both citalopram and fluoxetine have been shown to pharmacologically inhibit cardiac potassium channel current, which is imperative for normal ventricular repolarization.

During the past 5 years, there has been an increase in the utilization of escitalopram, the S-enantiomer of citalopram, for depression. Although there are published reports of escitalopram overdose, there is limited information on whether escitalopram causes QT prolongation and cardiac arrhythmias. Three cases of QT prolongation with escitalopram overdose have been reported [8–10]. In a toxicology review of 79 patients, van Gorp et al. demonstrated that QT prolongation along with serotonin toxicity and bradycardia was consistent clinical manifestation of escitalopram overdose [11].

We believe that this report not only offers additional insight into the potential for escitalopram to cause LQTS but also describes a unique case of drug-induced QT prolongation superimposed upon a patient with previously undiagnosed congenital LQTS. Given the frequency with which depression is currently diagnosed, along with the potential risk of SSRIs to induce cardiac arrhythmias coupled with the fact that most patients with congenital LQTS may initially appear asymptomatic, consideration should be given to a screening ECG prior to the initiation of SSRIs such as escitalopram. Indeed, particular caution should be taken when initiating such medications in patients deemed at risk for QT prolongation based upon serum electrolyte derangements, prior medical history, family history, or recent drug exposures.

## Learning Objective

An escitalopram overdose may be associated with serious cardiac arrhythmias, including QT prolongation and torsades de pointes. Prior to initiating therapy with selective serotonin reuptake inhibitors, health care providers should obtain a detailed prior medical history to uncover patients at high risk for previously undiagnosed QT prolongation. A screening electrocardiogram may be warranted prior to beginning a selective serotonin reuptake inhibitor.
